# Sex-Related Differences in the Prevalence of Classical, Non-Classical Risk Factors and Management of the Chronic Coronary Syndrome

**DOI:** 10.3390/jcm12237320

**Published:** 2023-11-26

**Authors:** Paweł Muszyński, Elżbieta Pawluczuk, Marta Pasławska, Maciej Kowalczuk, Julia Kozakiewicz, Natalia Sot-Muszyńska, Marcin Kożuch, Sławomir Dobrzycki

**Affiliations:** 1 Department of Invasive Cardiology, Medical University of Bialystok, M. Skłodowskiej-Curie 24A, 15-276 Bialystok, Poland; 2 Department of General and Experimental Pathology, Medical University of Bialystok, Mickiewicza 2C, 15-230 Bialystok, Poland; 3 Department of Cardiology, Lipidology and Internal Diseases, Medical University of Bialystok, Żurawia 14, 15-569 Bialystok, Poland; 4 Department of Pediatrics, Endocrinology, Diabetology with Cardiology Divisions, Medical University of Bialystok, J. Waszyngtona 17, 15-274 Bialystok, Poland; 5 Department of Internal Medicine with a Cardiological Profile, PCK Municipal Hospital in Bialystok, Sienkiewicza 79, 15-003 Bialystok, Poland

**Keywords:** myocardial ischemia, risk factors, coronary artery disease, sex-related differences

## Abstract

(1) Background: Coronary artery disease (CAD) remains the leading cause of death in both sexes. The male sex is considered a classical atherosclerosis risk factor, whereas females should be protected by hormonal effects until menopause. Although there are known differences in the development, type, and prognosis of chronic coronary syndrome (CCS) between both sexes, there are no differences in approach in the guidelines. (2) Methods: The sex-related differences in CAD risk factors, treatment, echocardiographic, and angiographic results were assessed among 3291 patients with CCS. (3) Results: Women were older and had a higher prevalence of hypertension, dyslipidaemia, and diabetes mellitus than men. Women were more often treated conservatively than men. There was no difference in the use of beta-blockers and statins among the sexes. The LDL cholesterol goal was less frequently reached by women. Women were treated less often with aspirin than men, but they were treated more often with angiotensin receptor blockers than men. The left ventricle ejection fraction was higher among females. The number of obstructed vessels was higher in men. (4) Conclusions: Women may be more exposed to the risk factors of CAD than men. Men are diagnosed with CAD earlier, and their prevention and therapy are more efficient.

## 1. Introduction

Coronary artery disease (CAD) can be defined as a pathological process characterized by atherosclerotic plaque accumulation in the epicardial arteries, whether obstructive or non-obstructive. It is a leading cause of death worldwide, and it resulted in 8.9 million deaths in 2015 [1,2]. However, there is a global decrease in the CAD death rate due to better diagnostics and earlier invasive treatment.

As opposed to men, CAD in women is often presented with atypical symptoms, and it makes the diagnostic process much more challenging. Women may experience milder symptoms, such as fatigue, shortness of breath, or nausea, rather than classical chest pain [3]. Moreover, women are diagnosed with CAD at an older age and suffer from many comorbidities. Unfortunately, there is still a lack of information regarding sex-specific CAD development characteristics. Many suggest that female patients deal with ischemia and non-obstructive coronary artery disease (INOCA) more often than men [4]. It is connected with a lower rate of CAD treated invasively among women than among men. Moreover, it is also connected with the misconception that no visible coronary artery obstruction in coronagraphy excludes the presence of CAD [4,5,6]. A new approach suggests that the occurrence of INOCA in women may be related to microvascular coronary artery disease and should be the motive for the intensification of pharmacotherapy [6]. Furthermore, it is suggested that a lack of estrogens in the perimenopausal period may lead to the impairment of nitric oxide synthase functioning and as a result, it may cause vasoconstriction [7].

There are many factors that increase the risk of coronary artery disease; some of them can not only be a target for prevention but also a motive for the initiation of the diagnostic process. The main factors are presented in Table 1.

Although CAD affects both sexes, some specific differences are connected with symptoms and the characteristics of risk factors and outcomes between men and women. According to some authors, the reasons for the differences may be different sex chromosomes, gene expression, and hormone levels [32,33,34]. Addressing these risk factors is fundamental for both the prevention and management of CAD. Hence, in clinical practice, the emphasis should be placed on early diagnostics and prophylactic intervention in women.

As our understanding of CAD continues to expand and evolve, ongoing research strives to refine diagnostic criteria, improve risk stratification, and develop innovative therapeutic strategies, ultimately contributing to more efficient prevention and treatment approaches for this widespread cardiovascular condition.

In this study, we aimed to increase the knowledge about sex-associated differences in the Polish population with CAD to enable better prevention and treatment.

## 2. Materials and Methods

### 2.1. Data Collection

The analysis was performed retrospectively according to the data collected by the Invasive Cardiology Department of the Medical University of Bialystok, Poland, from 27 December 2007 to 30 May 2016. In total, 3921 patients out of 11,155 total patients were included in the analysis. They were admitted to invasive diagnostics or invasive treatment (continuation of percutaneous treatment or as a decision of the multidisciplinary heart team).

The inclusion criteria consisted of the diagnosis of chronic coronary syndrome (CCS—I.25 ICD-10 code) at discharge after coronarography or percutaneous intervention. The exclusion criteria consisted of unstable angina (UA—I20), myocardial infarction (MI-I21), and acute heart failure.

Our study investigated the CAD risk factors, treatment, echocardiographic, and angiographic results and analyzed diversification according to the patient’s sex.

Incomplete coronarography (single vessel; 88.55 ICD 9 code) and echocardiography results were excluded from the analysis.

### 2.2. Statistical Analysis

Continuous variables are expressed as mean ± standard deviation. Categorical variables are expressed as percentages (number of patients). The adequacy of all parameters to normal distribution was tested using the Kolmogorov–Smirnov test. To compare parametric continuous variables, we used the Student’s *t*-test to compare nonparametric continuous variables, the Mann–Whitney U-test to compare categorical variables, and the chi-square test to analyze variances (ANOVA). Statistics were performed using Statistica 13.3. A *p*-value ≤ 0.05 was considered significant.

## 3. Results

### 3.1. Study Population

The study population consisted of 3291 patients. They were divided into two groups: men (*n* = 2242; 68.125%) and women (*n* = 1049; 21.875%). The average age of the patients was 66.71 ± 10.05 years. Moreover, the analyzed group was characterized by a high prevalence of risk factors for CAD, such as overweight (78.76%), hypertension (80.09%), diabetes mellitus (28.53%), dyslipidemia (64.57%), and chronic kidney disease (12.22%) (Table 2).

### 3.2. Classical Risk Factors

Women were characterized by more advanced age (69.62 ± 9.12 y. vs. 65.35 ± 10.19 y.; *p* < 0.0001), and 31.74% of them were older than 75 years in comparison to 20.65% of men. The age difference was 4.27 years.

We observed that women had lower weight than men (74.11 ± 12.83 kg vs. 85.41 ± 14.39 kg; *p* < 0.0001) but there was no difference in body mass index (BMI) (28.84 ± 4.78 vs. 28.58 ± 4.26) and occurrence of overweight (77.98% vs. 79.13%).

There was a higher prevalence of diabetes mellitus (32.41% vs. 26.85%; *p* = −0.001) and higher fasting blood glucose concentration (117.53 ± 45.24 mg/dL vs. 112.17 ± 37.58 mg/dL; *p* = 0.0004) among females (Table 2). Dyslipidemia was also found more frequently in females than in males (69.02% vs. 62.49%.; *p* = 0.0003). Furthermore, the total cholesterol level (TC) (173.57 ± 42.57 mg/dL vs. 162.83 ± 41.87 mg/dL; *p* < 0.0001), low-density lipoprotein cholesterol (LDL-C) (100.34 ± 37.51 mg/dL vs. 95.13 ± 35.73 mg/dL), and high-density lipoprotein cholesterol (HDL-C) (50.54 ± 20.28 mg/dL vs. 46.16 ± 19.78 mg/dL) was higher among women (Table 2). The concentration of triglycerides (TG) (131.14 ± 66.37 mg/dL vs. 137.86 ± 100.88 mg/dL; *p* = 0.049) was higher among males.

Hypertension also occurred more frequently among females (84.56% vs. 77.16%; *p* = 0.008). Systolic blood pressure was higher among women (133.97 ± 23.06 mmHg vs. 132.21 ± 20.27 mmHg; *p* = 0.03), but diastolic blood pressure was higher among males (75.02 ± 13.85 mmHg vs. 76.64 ± 12.95 mmHg; *p* = 0.001) (Table 2).

### 3.3. Non-Classical Risk Factors

Asthma (2.96%; vs. 0.76%; *p* < 0.0001), chronic kidney disease (14.30% vs. 11.24%; *p* = 0.0125), hyperfibrinogenemia (78.84% vs. 70.21%; *p* = 0.0018), hypothyroidism (4.39% vs. 1.25%; *p* < 0.0001), and rheumatoid arthritis (1.05% vs. 0.18%; *p* = 0.0006) occurred more frequently among women than among men (Table 3). A higher prevalence of hyperthyroidism (3.81% vs. 2.72%; *p* = 0.090) occurred among females.

In contrast, chronic obstructive pulmonary disease (COPD) (3.81% vs. 5.84% *p* = 0.015), history of past MI (36.99% vs. 48.17%; *p* < 0.0001), peripheral artery disease (8.48% vs. 10.75%; *p* = 0.044), anemia (18.21% vs. 31.53%; *p* < 0.0001), and hyperuricemia (14.39% vs. 18.64%; *p* = 0.003) were more frequent in males than in females (Table 3). No statistical differences were observed in obstructive sleep apnea and history of past stroke occurrence.

The concentration of uric acid (5.62 ± 1.81 mg/dL vs. 6.14 ± 1.69 mg/dL; *p* < 0.0001), hemoglobin (13.04 ± 1.32 g/dL vs. 14.00 ± 1.40 g/dL; *p* < 0.0001), and eGFR (69.58 ± 24.31 mL/min/1.73 m^2^ vs. 86.09 ± 31.67 mL/min/1.73 m^2^; *p* < 0.0001) values were higher in men.

The frequency of atrial fibrillation (11.92% vs. 12.49%; *p* = 0.64) and heart rate values remained comparable in both sexes (Table 3).

### 3.4. Treatment

Aspirin and other antiplatelet agents were taken by women less frequently than by men. Statins were taken comparably often by both sexes (91.23% vs. 91.39%; *p* = 0.88), as well as beta-blockers (92.66% vs. 92.64%; *p* = 1). However, females received simvastatin more often than atorvastatin or rosuvastatin (*p* = 0.011). Angiotensin-converting enzyme inhibitors (ACE-I) were more often used by males, whereas angiotensin receptor blockers (ARB) were more frequently prescribed to females (Table 4).

Both coronary artery bypass grafting (CABG) and percutaneous coronary interventions (PCI) were more often used as treatment options for males. Conservative treatment was more common among females (32.02% vs. 40.51%; *p* < 0.0001) (Table 4).

**Figure 1:**
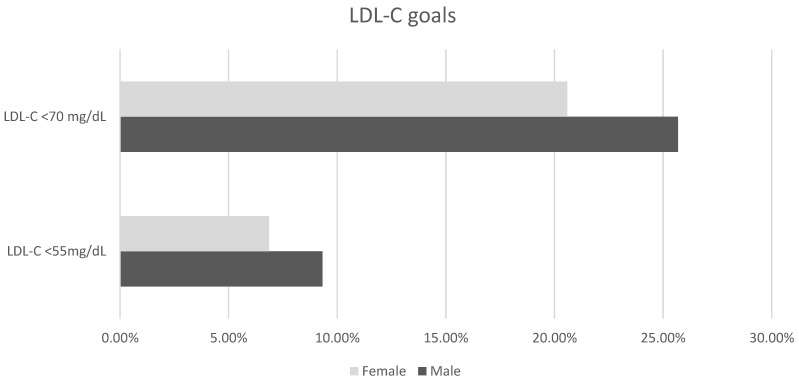
The difference between males and females in reaching low-density lipoprotein cholesterol goals; *p* < 0.05.

Women’s blood pressure was controlled less effectively than men’s (63.20% vs. 68.82%; *p* = 0.001). LDL-C under 70 mg/dL (20.59% vs. 25.69%; *p* = 0.001) and under 55 mg/dL (6.863% vs. 9.322%; *p* = 0.019) were less effectively achieved in females (Figure 1). HDL-C above 45 mg/dL occurred more often among women than above 40 mg/dL among men (41.7% vs. 36.03%; *p* = 0.002).

### 3.5. Echocardiography

Complete echocardiography was performed on 1352 patients. Left ventricular ejection fraction was higher among females (52.04 ± 10.62% vs. 47.89 ± 11.27%; *p* < 0.0001). All analyzed dimensions were higher among males (*p* < 0.0001); however, after indexing body surface area (BSA), the relation changed towards higher values in females (Table 5).

### 3.6. Angiographic Results

Complete coronarography was performed on 2704 patients (Table 6). Non-significant obstruction was more common among females (39.75% vs. 29.68%). The severity of CAD (including the number of obstructed vessels) was higher among males (<0.0001).

The total number of patients with chronic total occlusion (CTO) was 495 (18.3%). The occurrence of CTO was higher among males (12.53% vs. 21.08%; *p* < 0.0001).

## 4. Discussion

There are sex-related differences in pathogenesis and atherogenesis potency related to risk factors of chronic coronary syndrome. CCS occurs at an advanced age in women, mainly postmenopausal (in our study, the difference between sexes was equal to 4.27 years). This period of life is associated with a higher prevalence of many CAD risk factors, as confirmed in our study. It may predispose to widespread development of atherosclerosis, s more likely in the form of microvascular disease [35,36]. Females deal not only with obstructive CAD but also with non-plaque-related CAD [37]. Women have a higher rate of connection to myocardial bridging CAD, which causes coronary artery obstruction by stress-related vasoconstriction [36].

Importantly, the risk factors for obstructive and non-obstructive CAD are similar, and the best results in decreasing CAD-related mortality can be achieved using primary prevention. In addition to typical CAD risk factors, many other diseases are related to higher cardiovascular risk. While estimating risk using tools like the SCORE scale or Framingham scale, higher risk has to be assumed, and a preventive treatment must be applied earlier. In our study, we noticed a higher prevalence not only of hypertension, dyslipidemia, and diabetes mellitus but also of asthma, chronic kidney disease, hyperthyroidism, hypothyroidism, and rheumatoid arthritis in women. Men were diagnosed with COPD, past MI, peripheral artery disease, anemia, and hyperuricemia more often than women. This is important because diabetes mellitus, hypertension, and smoking have a stronger association with obstructive CAD among women than men [38]. Furthermore, when four cardiac risk factors were simultaneously present, the risk of obstructive CAD was nearly two times higher in women than men (OR 4.30 vs. OR 2.26; *p* < 0.001) [38]. In a larger meta-analysis including 9 686 513 participants (587 867 with atrial fibrillation—AF), AF was connected with an increased risk of ischemic heart disease (relative risk 1.61, 95% confidence interval 1.38 to 1.87) [31]. Bialystok Coronary Project results showed that atrial fibrillation occurs more often in males and is connected with non-obstructive coronary lesions [30]. 

In our study, the prevalence of classical and non-classical risk factors among both sexes explains nearly all cases of CAD. We were only unable to determine a noticeable CAD risk factor in 17 cases (0.52%).

Women with myocardial infarction suffered from radiation of the pain, palpitations, or dyspnea more often than men. Moreover, women were more likely to have myocardial infarction with non-obstructive coronary arteries (MINOCA) and were less often referred to cardiac rehabilitation after MI [39]. Women suffering from acute coronary syndrome (ACS) received primary PCI less often and had longer door-to-balloon time, resulting in higher 1-year mortality after STEMI than men [40].

Prevention is the most important tool to decrease cardiovascular mortality, but early diagnosis of CAD is equally significant. Many scientists suggest that due to less specified chest pain (as the primary symptom or dyspnea and less specific ECG changes), females may benefit from higher rates of cardiac stress tests and myocardial perfusion scintigraphy [36,41,42]. While considering female patients, we must change the stress test interpretation paradigm. In this interpretation paradigm, we interpret a positive stress test that is not confirmed with coronarography as a false positive result. It is known that in such cases, there is a high probability of dealing with ischemia and non-obstructive coronary artery disease, and pharmacological treatment should be intensified [4].

The authors suggest that the diagnostic process of chronic coronary syndrome should start from the assessment of pre-test probability and clinical likelihood of coronary artery disease based on age, sex, symptomatic presentation, risk factors, and additional test results [2]. If CAD cannot be excluded by clinical assessment alone, non-invasive functional imaging for myocardial ischemia or coronary computed tomography angiography (CTA) is recommended [2]. Invasive coronary angiography can be an alternative to other tests in patients with a high clinical likelihood and severe symptoms despite optimal medical therapy or typical angina at a low level of exercise [2]. The proposed approach focuses on the search for obstructive coronary artery disease and must be continued after the exclusion of severe vessel stenosis [4]. The important steps include considering coronary vasospasm or coronary microvascular dysfunction [4]. The adenosine-mediated coronary flow reserve, index of microvascular resistance, and hyperemic microvascular resistance should be measured in patients with angina or ischemia with no obstructive coronary disease because it allows proper pharmacological treatment choice or invasive treatment with coronary sinus reducer [4].

In a study involving 642 records from consecutive patients at the catheter laboratory, <30% stenosis or no lesions were found in 38.5% of patients and existed more frequently in females. In our study, non-significant lesions occurred in 42.3% of patients, predominantly in women [43]. Regarding the invasive treatment of coronary artery disease, in our study, both PCI and CABG were less often used in women. Similar data were also published in other studies [5,6,44]. In females, obstructive coronary artery disease invasive treatment, especially with coronary artery bypass graft, presented poorer results in comparison to males [36,45].

Both guidelines regarding prevention (2021) and ACS (2023) state that there is a need for sex-specific awareness campaigns with the aim of reducing sex disparities in research and clinical care [46,47]. The authors state that, while treating ACS, both genders receive benefits from invasive and non-invasive management strategies. However, there is possible gender/sex bias in past trials, and in order to assure quality evidence-based care for women, patient recruitment should be as similar to the real-world population as possible. Female involvement in trials must be assured in order to increase the knowledge regarding the optimal treatment of ACS [46]. In our opinion, similar efforts must also be made regarding CCS.

For example, in SYNTAX II, the PCI arm of SYNTAX I and CABG arm of SYNTAX I (94.9%), the majority of the subjects were male (93.2%, 93%, and 94.9%, respectively). The female sex had no impact on 5-year all-cause death (RR 0.867 [0.414–1.818] *p* = 0.71), but the population was extremely limited (female *n* = 31 in SYNTAX II); thus, the results could not be generalized [48].

In our study, during the analysis of medications/drugs that affect prognosis, we noticed that there was no statistically significant difference in the use of statins between males and females, but there were still significantly higher LDL-C levels in women. This may be explained by higher prescriptions of weaker statins. These results are supported by Nanna et al., who analyzed the effectiveness of statin treatment in accordance with the guidelines of the American College of Cardiology/American Heart Association (2013). The study showed that statins were prescribed less often to women (67.0% vs. 78.4%; *p* < 0.001), and women were treated less often with recommended intensity in comparison with men (36.7% vs. 45.2%; *p* < 0.001). Furthermore, there was a tendency among doctors not to offer statins to women. When treatment with statins was offered, these drugs were often declined by women [49]. Moreover, females lacked confidence towards statins’ safety (55.2% vs. 47.9%; *p* < 0.001) and effectiveness more frequently in comparison to men (73.2% vs. 68.0%; *p* < 0.001) [49]. In the analysis of 685 patients who were prescribed statins in monotherapy, initially, the women had higher TC, LDL-C, and HDL-C. However, there was no difference in the proportion of men and women who achieved LDL-C ≤ 2.5 mmol/L at the follow-up [50]. Unfortunately, even though statins reduced the incidence of hospitalization for CAD and/or non-hemorrhagic cerebrovascular disease, adherence to statin therapy was extremely low and declined shortly after initiation. At the 1-year follow-up, the proportion of adherent subjects was equal to 28% in women and 38% in men [51].

Although antiplatelet agents improve prognosis in both sexes, we observed that aspirin and other antiplatelet agents were less often used among females. We also noticed that females were treated with ACE-I less often, while ARB were more preferred in this group. Ferrari et al. also noticed that females were treated less often with aspirin, ACE-I, and lipid-lowering drugs [42,52]. Moreover, Hemal et al. observed in the PROMISE trial that females received aspirin and ACE-I less often, whereas they had higher doses of statins and b-blockers [42]. To sum up, these studies support the results of our research and emphasize that although pharmacological treatment is essential in females, they tend to receive optimal CAD therapy less frequently.

During our study, the low-density lipoprotein cholesterol treatment goal for patients with cardiovascular disease was 70 mg/dL, but even such a liberal target was reached only by approximately 20% of patients at the point of admission for coronarography.

The European Society of Cardiology (ESC) and European Atherosclerosis Society (EAS) Guidelines regarding the management of dyslipidemia initiated more restrictive goals for patients with very high cardiovascular risk, targeting achievement of at least a 50% reduction from baseline and an LDL-C < 1.4 mmol/L (<55 mg/dL), with Class I recommendations, level of evidence A for secondary prevention and with Class I recommendations, level of evidence C for primary prevention [53]. Additional tools for the reduction of cardiovascular risk were also introduced in the form of recommendations for ezetimibe and proprotein convertase subtilisin/kexin type 9 inhibitors [53].

Furthermore, the Task Force for Cardiovascular Disease Prevention in Clinical Practice, including representatives from ESC and the European Association of Preventive Cardiology (EAPC), set a new goal of <1.0 mmol/L (40 mg/dL) for patients with atherosclerotic cardiovascular disease (ASCVD), e.g., those who experience a second vascular event within 2 years while receiving the maximum, tolerated statin-based therapy [47].

Chronic Ischemic Cardiovascular Disease Long-Term (CICD-LT) registry data shows that women with CCS have higher cardiovascular mortality than men (2.0% vs. 1.3%, *p* = 0.02) [54]. Furthermore, 66.5% of enrolled patients do not have optimal low-density lipoprotein-cholesterol levels [54]. The opposite results can be obtained from the PRESAGE registry. At 12-month follow-up, the composite endpoint was more frequently reached in men (7.4% vs. 10.2%; *p* < 0.001) [5]. However, in multivariable analysis, sex was not an independent predictor of the composite endpoint [5]. Additionally, in a 17-year prospective cohort study, despite the similar prescriptions of statins, antiplatelets, and beta-blockers, major cardiovascular events (acute myocardial infarction, stroke, or cardiovascular death) were more frequent among women [55].

Generally, females have higher LVEF than men (a normal range for women is 52–72% vs. 54–74% in men) [56]. In our study, in subjects with CCS, LVEF was also higher in women (52.04% vs. 47.89%). However, some authors suggest that even when LVEF is within the normal range, global longitudinal strain declines with age only in females [57].

Wu et al. suggest that sex is an important determinant of cardiac structure and function. Women in pre-menopause are better protected against cardiac hypertrophy compared with men because of hormonal influence. After menopause, this protection is reduced or could be partially restored after estrogen replacement therapy [58].

Left ventricle diameters are characterized by lower normal values for women [56]. However, there is a strong impact on body size that can be defined by BSA. Higher BSA in men corresponds with a higher diameter of most heart structures. In our study, all dimensions were higher in men, but after BSA indexing, the relation changed to the opposite [59].

## 5. Conclusions

The prevalence and impact of unclassical risk factors on coronary artery disease are very high, especially among females. There is a need to increase practitioners’ knowledge concerning unclassical risk factors and their role in CAD prevention. Tailoring interventions based on the patient’s individual risk factors, considering hormonal influences in women, and exploring novel therapeutic options should become integral components of CAD management in males and females. Additionally, a wide range of risk factors makes CAD prevention not only a subject of interest to cardiologists but also to other specialists. It is crucial for healthcare improvement to prepare more personalized guidelines concerning sex-related differences for diagnosing and treating CAD. Moreover, female patients require more intense primary and secondary prevention, as classical risk factors of CAD occur more frequently among them. Pharmacological treatment plays a crucial role and must be as intensive as the patient can tolerate.

## 6. Study Limitations

### 6.1. Study Design

The study was performed as a retrospective, observational analysis; the subjects originated from Eastern Europe, and all participants were Caucasian; thus, the results cannot be generalized to other ethnicities.

The study does not include follow-up, and the clinical endpoints, such as survival or combined long-term major adverse cardiac and cerebrovascular event rates, were not analyzed.

### 6.2. Data Collection

The retrospective aspect of the study, based on data acquired from medical documentation, does not allow for the inclusion of a variety of factors, such as autoimmunological diseases, Cushing disease, erectile disorders, depression, and risk factors unique to women, e.g., disorders related to pregnancy and reproduction, especially hypertensive disorders of pregnancy, gestational diabetes, and menarche/menopause [36,60]. Additionally, not all patients had complete echocardiography and coronarography performed during hospitalization.

## 7. Future Research Directions

We are planning to continue the research by including a population from 2016 to 2023 in order to evaluate the impact of changes in the guidelines made by ESC/EAS/EAPC. That would allow the analysis of trends in risk factor prevalence, as well as in treatment. Furthermore, evaluation of clinical endpoints by follow-up will be considered in order to analyze the longitudinal effect.

## Figures and Tables

**Table 1:** Risk factors of coronary artery disease; COPD—chronic obstructive pulmonary disease.

Risk Factors of Coronary Artery Disease		
Classical risk factor	Male	Dyslipidemia
	Hypertension	Diabetes Mellitus
	Age	Overweight
	Smoking *	Family history *
Non-classical risk factor	Asthma [8]	COPD [9]
	Obstructive sleep apnea [10]	Past stroke [11]
	Peripheral artery disease [12]	Chronic kidney disease [13,14]
	Anemia [15]	Hyperuricemia [16,17,18]
	Hyperfibrinogenemia [19]	Thyroid hormone dysfunction [20]
	Rheumatoid arthritis [21]	Psychiatric disorders [22] *
	Sexual disorders [23] *	Socioeconomic factors [24] *
	Pregnancy and reproduction-related disease [25,26] *	Liver disease [27] * Environmental factors and Pollution [28,29] *
	Atrial fibrillation [30,31]	

* Non-analyzed in the study.

**Table 2:** Classical risk factors: BMI—body mass index; TC—total cholesterol; LDL-C—low-density lipoprotein cholesterol; HDL-C—high-density lipoprotein cholesterol; TG—triglycerides; SBP—systolic blood pressure; DBP—diastolic blood pressure.

Classical Risk Factor	Total	Male	Female	*p*-Value
Sex (male)	68.125% (2242)	-	-	-
Age (above 75 y.)	25.06% (825)	20.65% (463)	31.74% (333)	<0.0001
Age [y.]	66.71 ± 10.05	65.35 ± 10.19	69.62 ± 9.12	<0.0001
Overweight	78.76% (2592)	79.13% (1774)	77.98% (818)	0.45
Weight [kg]	81.77 ± 14.80	85.36 ± 14.28	74.11 ± 12.83	<0.0001
BMI	28.67 ± 4.43	28.58 ± 4.26	28.84 ± 4.78	0.10
Diabetes Mellitus	28.53% (939)	26.85% (602)	32.41% (340)	0.001
Glucose [mg/dL]	113.88 ± 40.26	112.17 ± 37.58	117.53 ± 45.24	0.0004
Dyslipidemia	64.57% (2125)	62.49% (1401)	69.02% (724)	0.0003
TC [mg/dL]	166.25 ± 42.39	162.83 ± 41.87	173.57 ± 42.57	<0.0001
LDL-C [mg/dL]	96.79 ± 36.39	95.13 ± 35.73	100.34 ± 37.51	0.0001
HDL-C [mg/dL]	46.16 ± 19.78	44.12 ± 19.20	50.54 ± 20.28	<0.0001
TG [mg/dL]	135.72 ± 91.35	137.86 ± 100.88	131.14 ± 66.37	0.049
Hypertension	80.09% (2636)	77.16% (1730)	84.56% (887)	0.008
SBP [mmHg}	132.77 ± 21.21	132.21 ± 20.27	133.97 ± 23.06	0.03
DBP [mmHg]	76.13 ± 13.27	76.64 ± 12.95	75.02 ± 13.85	0.001

**Table 3:** Non-classical risk factors; COPD—chronic obstructive pulmonary disease; cut-off value for anemia was defined as hemoglobin levels <12.0 g/dL in women and <13.0 g/dL in men; for hyperuricemia, it was defined as serum uric acid >7.0 mg/dL for men and >6.0 mg/dL in women.

Non-Classical Risk Factor	Total	Male	Female	*p*-Value
Asthma	1.46% (48)	0.76% (17)	2.96% (31)	<0.0001
COPD	5.20% (171)	5.84% (131)	3.81% (40)	0.015
Obstructive sleep apnea	0.40% (13)	0.45% (10)	0.29% (3)	0.5
Past MI	42.90% (1412)	48.17% (1080)	36.99% (388)	<0.0001
Past stroke	5.47% (180)	5.53% (124)	5.34% (56)	0.82
Peripheral artery disease	12.94% (426)	10.75% (241)	8.48% (89)	0.044
Chronic kidney disease	12.22% (402)	11.24% (252)	14.30% (150)	0.0125
Creatinine [mg/dL}	1.03 ± 0.59	1.08 ± 0.66	0.94 ± 0.37	<0.0001
eGFR [mL/min/1.73 m^2^]	80.83 ± 30.51	86.09 ± 31.67	69.58 ± 24.31	<0.0001
Anaemia	27.28% (898)	31.53% (707)	18.21% (191)	<0.0001
Hemoglobin [g/dL]	13.70 ± 1.44	14.00 ± 1.40	13.04 ± 1.32	<0.0001
Hyperuricemia	17.32% (570)	18.64% (418)	14.39% (151)	0.003
Uric acid [mg/dL]	5.97 ± 1.74	6.14 ± 1.69	5.62 ± 1.81	<0.0001
Hyperfibrinogenemia	72.96% (2401)	70.21% (1574)	78.84% (827)	0.0018
Fibrinogen [mg/dL]	407.33 ± 91.79	400.09 ± 91.19	422.79 ± 91.20	<0.0001
Hyperthyroidism	3.07% (101)	2.72% (61)	3.81% (40)	0.090
Hypothyroidism	2.25% (74)	1.25% (28)	4.39% (46)	<0.0001
Rheumatoid arthritis	0.45% (15)	0.18% (4)	1.05% (11)	0.0006
Atrial fibrillation	12.31% (405)	12.49% (280)	11.92% (125)	0.64
Heart rate [beats per minute]	66.67 ± 12.78	66.53 ± 13.28	66.96 ± 11.62	0.36

**Table 4:** Treatment: CABG—coronary artery bypass grafting; PCI—percutaneous coronary interventions; ARB—angiotensin receptor blockers; ACE-I—angiotensin-converting enzyme inhibitors.

Treatment	Male	Female	*p*-Value
Invasive	67.98% (1524)	59.49% (624)	<0.0001
CABG	8.83% (198)	5.72% (60)	0.002
PCI	59.14% (1326)	53.77% (564)	0.004
Conservative	32.02% (718)	40.51% (425)	<0.0001
Aspirin	93.18% (2089)	90.66% (951)	0.01
Other antiplatelet agents	56.38% (1264)	51.19% (537)	0.005
Beta-Blockers	92.64% (2077)	92.66% (972)	1
ARB	9.99% (224)	21.73% (228)	<0.0001
ACE-I	80.37% (1802)	71.12% (747)	<0.0001
Statins	91.39% (2049)	91.23% (957)	0.88
Simvastatin	36.13% (810)	39.94% (419)	0.011
Atorvastatin	49.82% (1117)	44.5% (472)	
Rosuvastatin	5.44% (122)	6.10% (64)	
Fluvastatin	0% (0)	0.19% (2)	

**Table 5:** Echocardiography.

Echocardiography (*n* = 1352)	Male (*n* = 915)	Female (*n* = 437)	*p*-Value
Left ventricular ejection fraction [%]	47.89 ± 11.27	52.04 ± 10.62	<0.0001
≤40%	24.15% (221)	16.25% (71)	<0.0001
41–49%	20.98% (192)	12.12% (53)	
≥50%	54.86% (502)	71.62% (313)	
Left ventricular end-diastolic diameter [mm]	52.39 ± 6.61	47.94 ± 5.76	<0.0001
Right ventricular end-diastolic diameter [mm]	30.55 ± 3.57	28.43 ± 3.16	<0.0001
Left atrium [mm]	40.90 ± 5.97	39.02 ± 7.99	<0.0001
Ascending aorta [mm]	36.31 ± 4.16	33.45 ± 3.75	<0.0001
Intraventricular septum diameter [mm]	11.67 ± 1.71	11.18 ± 1.55	<0.0001
Posterior wall diameter [mm]	11.02 ± 1.37	10.65 ± 1.37	<0.0001
Values after indexing to Body Surface Area			
Body Surface Area	2.02 ± 0.19	1.81 ± 0.17	<0.0001
Left ventricular end-diastolic diameter [mm]	26.06 ± 3.78	26.81 ± 3.73	0.0002
Right ventricular end-diastolic diameter [mm]	15.12 ± 2.06	15.89 ± 2.05	<0.0001
Left atrium [mm]	20.24 ± 3.13	21.83 ± 4.92	0.0001
Ascending aorta [mm]	17.94 ± 2.35	18.69 ± 2.34	0.0003
Intraventricular septum diameter [mm]	5.80 ± 0.92	6.26 ± 1.02	<0.0001
Posterior wall diameter [mm]	5.47 ± 0.76	5.96 ± 0.91	<0.0001

**Table 6:** Angiographic results of 2704 patients.

Angiographic Results (Sample *n* = 2704)	Male (*n* = 1826)	Female (*n* = 878)	*p*-Value
Average obstructed major vessels number	1.14 ± 0.97	0.92 ± 0.94	<0.0001
Non-significant obstruction	29.68% (542)	39.75% (349)	<0.0001
Single-vessel	37.90% (692)	36.56% (321)	
Double-vessel	21.08% (385)	15.15% (133)	
Triple-vessel	11.34% (207)	8.54% (75)	
Presence of Chronic Total Occlusion *n* = 495 (18.3%)	21.08% (385)	12.53% (110)	<0.0001

## Data Availability

The data presented in this study are available on request from the corresponding author.

## Abbreviations

ACE-I	angiotensin-converting enzyme inhibitors
ACS	acute coronary syndrome
AF	atrial fibrillation
ARB	angiotensin receptor blockers
BMI	body mass index
BSA	body surface area
CABG	coronary artery bypass grafting
CAD	coronary artery disease
CCS	chronic coronary syndrome
COPD	chronic obstructive pulmonary disease
CTA	computed tomography angiography
CTO	chronic total occlusion
DBP	diastolic blood pressure
EAPC	European Association of Preventive Cardiology
EAS	European Atherosclerosis Society
ECG	electrocardiography
ESC	European Society of Cardiology
HDL-C	high-density lipoprotein cholesterol
INOCA	ischemia and non-obstructive coronary artery disease
LDL-C	low-density lipoprotein cholesterol
LVEF	left ventricular ejection fraction
MI	myocardial infarction
MINOCA	myocardial infarction with non-obstructive coronary arteries
PCI	percutaneous coronary interventions
SBP	systolic blood pressure
SCORE	systematic coronary risk evaluation
TC	total cholesterol
TG	triglycerides
UA	unstable angina
